# Disseminated Intravascular Coagulation in COVID-19 Setting: A Clinical Case Description

**DOI:** 10.7759/cureus.39941

**Published:** 2023-06-04

**Authors:** Tiago Ceriz, João Lagarteira, Sérgio R Alves, Andrés Carrascal, Rui Terras Alexandre

**Affiliations:** 1 Internal Medicine Department, Unidade Local de Saúde do Nordeste, Bragança, PRT; 2 Intensive Medicine and Emergency Department, Unidade Local de Saúde do Nordeste, Bragança, PRT

**Keywords:** hemorrhage, d-dimers, fibrinogen, disseminated intravascular coagulation, covid-19

## Abstract

Disseminated intravascular coagulation (DIC) is an acquired syndrome that can lead to catastrophic thrombosis and hemorrhage. In DIC, an uncontrolled release of pro-inflammatory mediators activates tissue factor-dependent coagulation. These changes cause endothelial dysfunction and increased depletion of platelets and clotting factors needed to control bleeding, which results in excessive bleeding. The clinical manifestations are microvascular thrombosis and hemorrhage, which cause severe organ dysfunction and worsening of organ failure. Its clinical management is challenging.

Coronavirus disease 2019 (COVID-19) is characterized mainly by respiratory manifestations. In severe cases, however, systemic inflammatory response syndrome can develop with cytokine release that leads to coagulopathy and DIC. Among patients with COVID-19, this complication occurs rarely, leading to death in the majority of cases. We describe the case of a 67-year-old woman with asthma and class 1 obesity, hospitalized with respiratory insufficiency after diagnosis of COVID-19, in whom DIC developed with hemorrhagic manifestations on Day 4 of hospitalization. In spite of poor prognosis and multiple complications throughout the 87 days of hospitalization, including 62 days in the ICU, this patient survived.

## Introduction

Coronavirus disease 2019 (COVID-19), caused by the severe acute respiratory infection coronavirus 2 (SARS-CoV-2), manifests with fever, dry cough, dyspnea, and fatigue; in some patients, pneumonia, sepsis, and acute respiratory distress syndrome develop [1]. Advanced age, cardiovascular disease, hypertension, diabetes, and chronic obstructive pulmonary disease (COPD) are among the most important independent predictors of death [2,3]. Of patients infected with SARS-CoV-2, 80% are asymptomatic or have only mild symptoms, but the other 20% become severely ill, and 2-5% die [4]. Among patients with COVID-19, DIC has been reported in 0.6% of patients who survive and in 71.4% of those who do not survive. Thus, DIC occurs in a high percentage of fatal cases, whereas few survivors have had DIC [4]. Preexisting increased plasmin activity, recognized in hypertension, diabetes, and cardiovascular disease, enhances the virulence and infectivity of SARS-CoV-2 by cleaving its spike protein [5]; thus, such activity is not only a determinant of the severity of COVID-19 but also may promote the development of DIC.

DIC, also called “consumption coagulopathy” or “defibrination syndrome,” is a systemic process in which thrombosis and hemorrhage can develop [6]. Acute (decompensated) DIC can develop when large amounts of tissue factor (or other procoagulant substances) accumulate in the blood over a brief period, with a significant generation of thrombin and rapid consumption of coagulation factors that outpaces their production. The fibrin degradation products, operating as pathologic anticoagulants and antiplatelet agents, interfere with both fibrin clot formation and platelet aggregation, which leads to severe bleeding and diathesis [6]. In addition, plasminogen activator inhibitor-1 suppresses fibrinolysis, which can result in blood clots.

No single laboratory marker to diagnose disseminated intravascular coagulation (DIC) has been identified [7]. Typical abnormalities in DIC are prolonged PT, prolonged aPTT, thrombocytopenia, elevated levels of fibrin-related markers, and reduced fibrinogen levels. To account for the various diagnosis limitations, and better assess these patients, scoring systems have been developed [8]. The International Society on Thrombosis and Haemostasis (ISTH) DIC score is widely used to diagnose overt DIC. To calculate the ISTH DIC score, four laboratory values (platelet count, PT, d-dimer or another fibrin degradation marker, and fibrinogen) are scored between 0 and 8. An ISTH DIC score of >5 supports the diagnosis of DIC [9].

The laboratory finding related to COVID-19 coagulopathy that is most frequently reported is an increase in plasma d-dimer levels [10-12]. Coagulation activation results in the formation of thrombi, which are dissolved through fibrinolytic activation. The degradation of stabilized fibrin polymer by plasmin causes the production of d-dimer in the blood [4]. This fact led to studies focusing on the relationship between elevated d-dimer levels and prognosis; concluding that a high concentration of d-dimer is an independent risk factor for poor outcomes [10-12]. However, if a large thrombus forms but is not dissolved (a serious complication), the increase in d-dimer may be mild. Thus, the degree of increase in d-dimer may not be correlated directly with the severity of disease [4].

In patients with COVID-19, the combination of increased d-dimer levels, low platelet counts, and (slightly) prolonged coagulation times resembles the abnormalities commonly seen in DIC, but COVID-19 coagulopathy differs in several ways from DIC that occurs with severe infections and sepsis. In sepsis-associated DIC, thrombocytopenia is more severe, levels of clotting factors are lower, and plasma concentrations of coagulation inhibitors are decreased [13]. Asakura and Ogawa also suggested that in COVID-19-related DIC, fibrinolysis is enhanced, whereas, in sepsis-related DIC, fibrinolysis is suppressed [4]. Thus, the relationship between coagulation dysfunction and prognosis in patients with COVID-19 is complex and might vary in different clinical studies. The reason for this discrepancy is unknown and might partly be attributable to patient selection, associated comorbidities, and pharmacologic treatments [14].

We describe the clinical case of a patient who developed DIC after her admission to the intensive care unit (ICU) for COVID-19 pneumonia.

## Case presentation

Herein, a 67-year-old woman was admitted to the emergency room with shortness of breath, wheezing, dry cough, chills, and muscular pain with 10 days of progression. Two days prior to admission, her symptoms worsened, and the patient tested positive for COVID-19.

**Figure 1:**
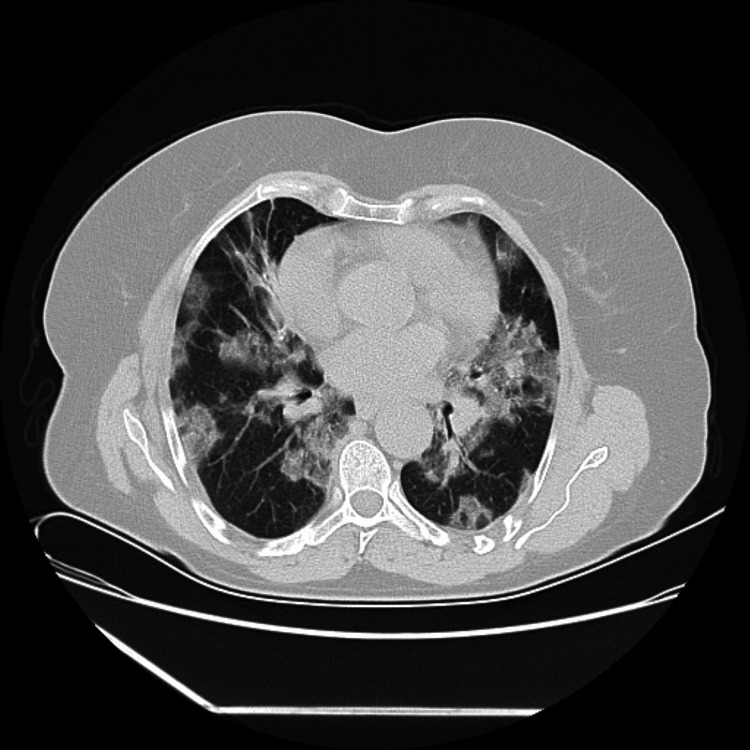
Computed tomographic scan of the thorax on the day of intensive care unit admission. Bilateral pneumonia from COVID-19, with diffuse bilateral subpleural ground glass opacities and fibrotic component.

The patient had class 1 obesity, vertigo, asthma since the age of 11, and a previous admission for viral tracheobronchitis with hypoxic respiratory insufficiency as comorbidities. At admission, objective examination revealed dyspnea and bilateral wheezing, as well as hypoxic respiratory insufficiency, tachypnea, and tachycardia. Regarding laboratory test results, the patient had hemoglobin of 14.1 (mg/dL), d-dimer of 482 (ng/mL), C-reactive protein of 11.1 (mg/dL), and an oxygen arterial pressure of 47.5 mmHg. The computed tomographic scan of the thorax showed severe bilateral pneumonia due to COVID-19, with diffuse bilateral subpleural ground glass opacities and fibrotic components, predominantly in superior lobes, affecting 60-70% of the total lung parenchyma, as shown in Figure 1.

**Figure 2:**
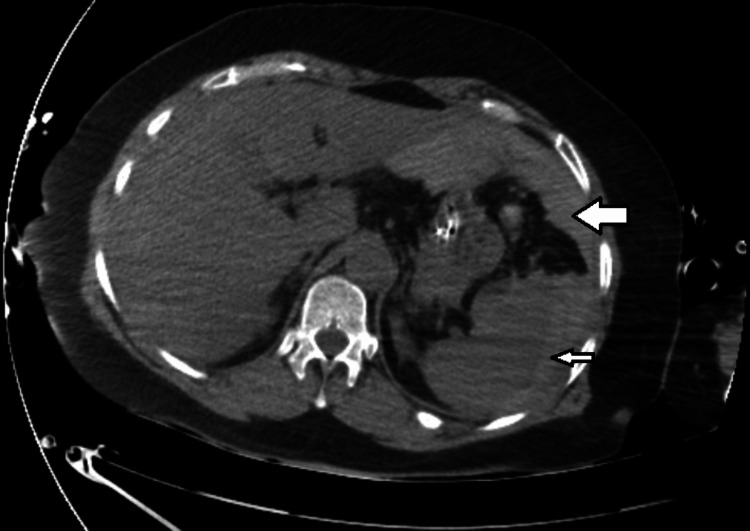
Computed tomographic scan of the abdomen on the 4th day in the intensive care unit. Hemoperitoneum (big arrow) due to splenic laceration (small arrow)

The patient was then admitted to the internal medicine ward, but ventilatory function progressively worsened with severe hypoxic respiratory insufficiency, and the patient was transferred to the intensive care unit the same day. In the next few days, the patient's clinical condition kept deteriorating and on Day 4 of hospital admission, the patient needed orotracheal intubation and prone positioning for better ventilatory dynamics. Later that day, the patient developed hemoperitoneum due to splenic laceration (Figure 2), with a loss of 6 grams of hemoglobin and hemorrhagic shock. The splenic laceration was probably due to increased abdominal pressure in prone positioning.

Patient stabilization required protamine sulfate for hypocoagulation reversal, tranexamic acid, and transfusion of 2 units of erythrocytes; these were followed by 1 unit of pooled platelets and 3 units of plasma. Despite clinical improvement, platelet and fibrinogen levels kept decreasing, and transfusions were ineffective. By Day 5, a massive airway hemorrhage originating in the right medial lobe developed spontaneously; selective intubation with a double lumen tube was performed to ventilate the left lung and inferior right lobe in order to insulate the hemorrhagic focus. Multiorgan dysfunction syndrome was manifested by macrocirculatory and microcirculatory dysfunction, hepatic dysfunction (total bilirubin level: 2 mg/dL), renal dysfunction (severe acidemia and need for renal substitution therapy), ventilatory dysfunction (severe bronchospasm and SARS), and hematologic dysfunction, with thrombocytopenia (platelet level: 55,000/mL), coagulopathy, and hypofibrinogenemia (fibrinogen level: 105 mg/dL). At this point, the d-dimer level was 341 ng/mL. On the basis of the clinical manifestations and laboratory results, and because the calculated ISTH score was >6 (ISTH score for DIC diagnosis is 5), the diagnosis of DIC was confirmed. The previous supportive measures with red blood cell, platelet, and plasma transfusion, as well as fibrinogen supplementation, were maintained until clinical stabilization was achieved. 

Tables 1-2 list some of the major events that occurred during the patient’s hospitalization and the gasometric and laboratory values at those times.

**Table 1:** Gasometric measurements in key events during patient’s hospitalization CID: clinical infectious disease; ER: emergency room; FiO2: fraction of inspired oxygen; HCO3: bicarbonate; ICU: intensive care unit; OTE: oxygen transfer efficiency; PCO2: partial pressure of carbon dioxide; PO2: partial pressure of oxygen.

Event	pH	pCO_2_ (mm Hg)	pO_2_ (mm Hg)	HCO_3_ (mmol/L)	Lactate (mmol/L)	Na^+^ (mmol/L)	K^+^ (mmol/L)	pO_2_/FiO_2_ ratio	Day
ER admission	7.43	33.9	47.5	22.6	0.9	140	3.9	226	1
ICU admission (O_2_ rate 15 L/h)	7.42	34.0	54.6	22.3	1.3	140	4.1	68	1
OTE testing	7.47	32	77.3	23	2.7	141	3.2	119	4
CID (airway hemorrhage)	7.20	62	75	23.9	1.7	139	3.3	75.2	5
Left hemopneumothorax	7.19	60	52	24	3.2	141	3.4	65	10
Right pneumothorax	7.34	67.4	70	25.4	4.2	143	3.5	70	16
Necrotizing pneumonia	7.36	49.8	89.1	27.8	1.3	151	3.7	78	20
Spontaneous ventilation, 31% FiO_2_	7.43	45	92.7	29.7	2.7	137	4	249	41
Discharge from ICU to internal medicine ward (O_2_ rate: 3 L/h)	7.43	37.3	125	24.7	1.4	140	4.3	390	62

**Table 2:** Laboratory parameters in key events during patient’s hospitalization apTT: activated thromboplastin time; DIC: disseminated intravascular coagulation; enx: treated with enoxaparin; Hb: hemoglobin; ICU: intensive care unit; INR: international normalized ratio; PCR: polymerase chain reaction; PT: prothrombin time.

Event	Hb (mg/dL)	Platelets (×10^9^/L)	INR	PT (sec)	apTT (sec)	Fibrinogen (g/L)	d-dimer (ng/mL)	PCR (mg/dL)	Day
ICU admission	14.1	180	1.28	13.9	30.1	401	482	11.1	1
Hemoperitoneum (splenic laceration)	7.6	146	1.44	—	—	320	421	0.4	4
DIC	7.0	55	1.25	14.1	32.5	105	341	1.08	5
Left pneumothorax	7.2	31	1.17	—	33.1	147	2100	2.50	10
Left hemopneumothorax	6.2	42	1.22	—	23.3	181	4046	1.65	13
Right pneumothorax	8.0	60	1.19	—	22	261	3850	2.8	16
Necrotizing pneumonia	9.7	87	1.36 (enx)	—	23.6 (enx)	2470	2040	4.97	20
Perisplenic abscess	8.3	132	1.42 (enx)		25.8 (enx)	—	1038	13.5	27
Hospital Discharge	9.5	412	1.18	12.9	30.7	—	—	0.5	87

**Figure 3:**
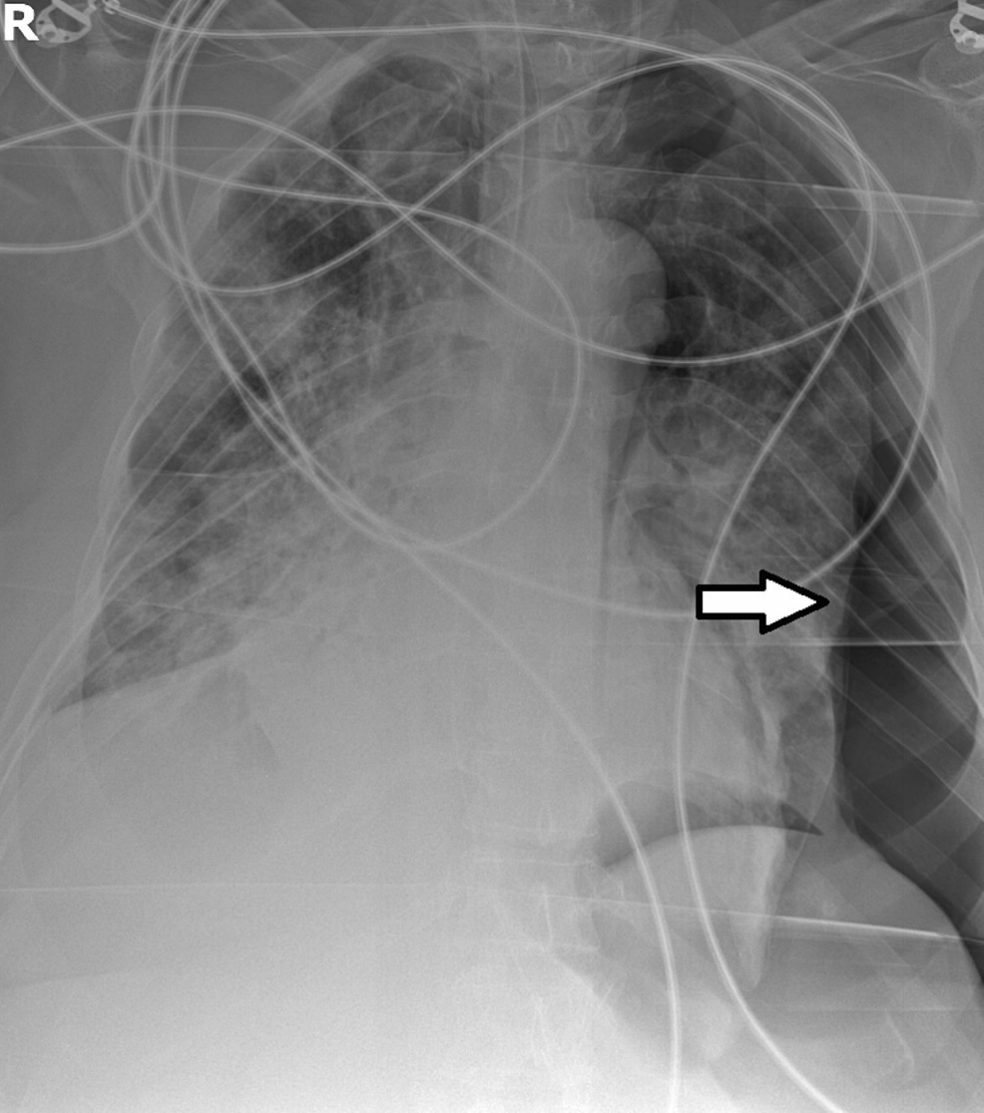
Radiograph of the thorax on the 10th day in the intensive care unit Left pneumothorax (arrow) is evident.

**Figure 4:**
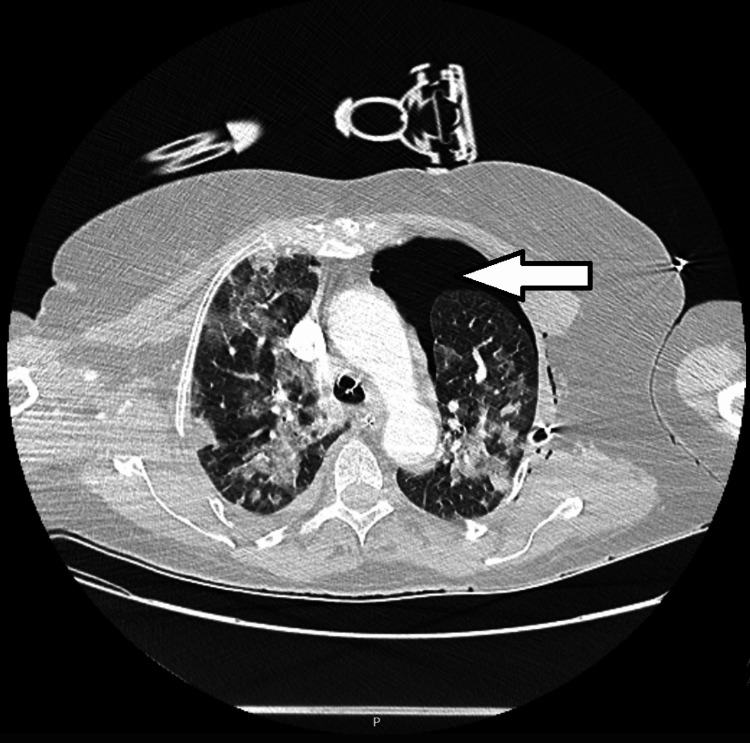
Computed tomographic scan of the thorax on the 10th day in the intensive care unit Left pneumothorax (arrow) and diffuse bilateral subpleural ground glass opacities with fibrotic components are evident.

At the end of Day 9 in the ICU, after two episodes of atrial fibrillation with rapid ventricular response that were treated with electrical cardioversion, the patient was stabilized. The hemorrhagic complications required transfusions of 5 units of red blood cells, 3 units of pooled platelets, 3 units of plasma, and 10 g of fibrinogen supplementation in total. At this time, DIC was considered resolved (ISTH score: <5), and bleeding manifestations had ceased; however, levels of platelets and d-dimer worsened (to 31,000/mL and 2100 ng/mL, respectively). Fibrinogen levels recovered somewhat (to 137 mg/dL) and no new hemorrhagic or thrombotic complications developed. On Day 10, however, a rapid decline in clinical condition and ventilatory function led to the diagnosis of left pneumothorax (Figures 3-4), complicated with subcutaneous emphysema and later with hemothorax on Day 13.

**Figure 5:**
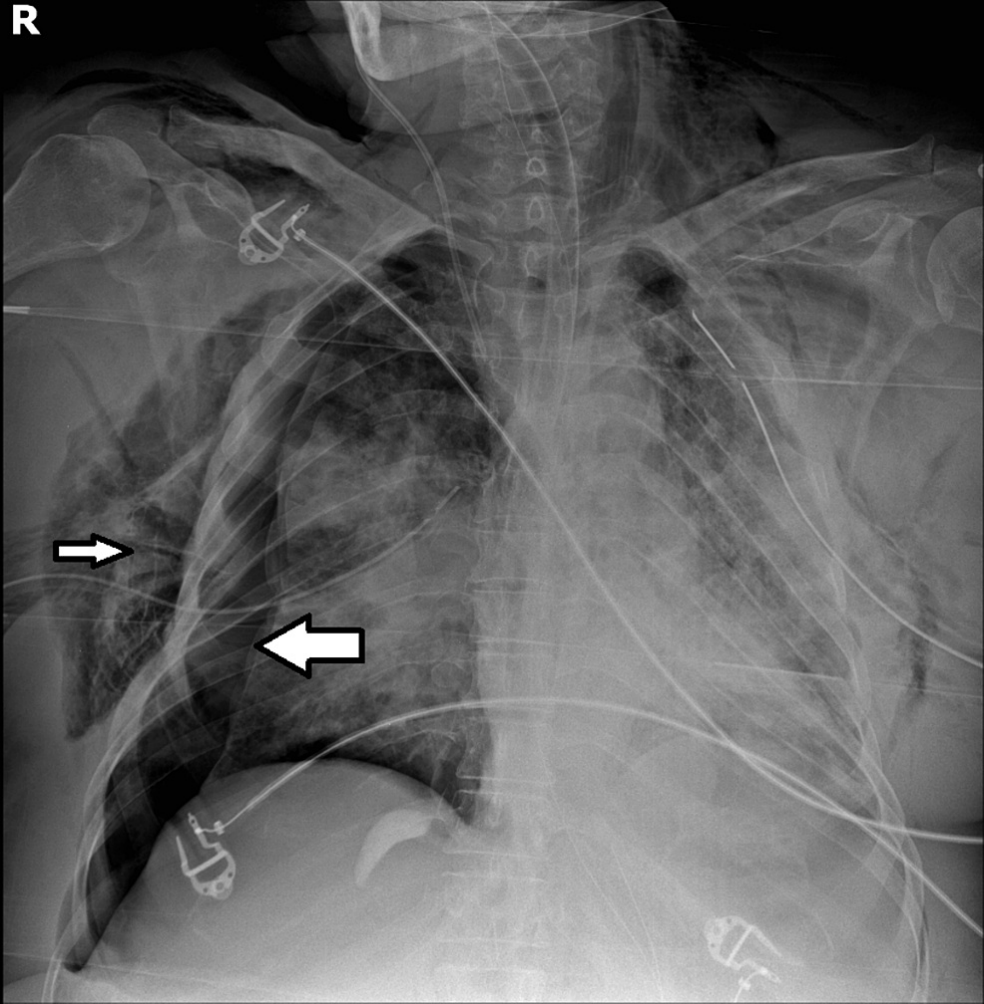
Radiograph of the thorax on the 16th day in the intensive care unit Right pneumothorax (big arrow) and subcutaneous emphysema (little arrow) are evident.

On Day 16, right pneumothorax was diagnosed and was also complicated with subcutaneous emphysema (Figure 5).

**Figure 6:**
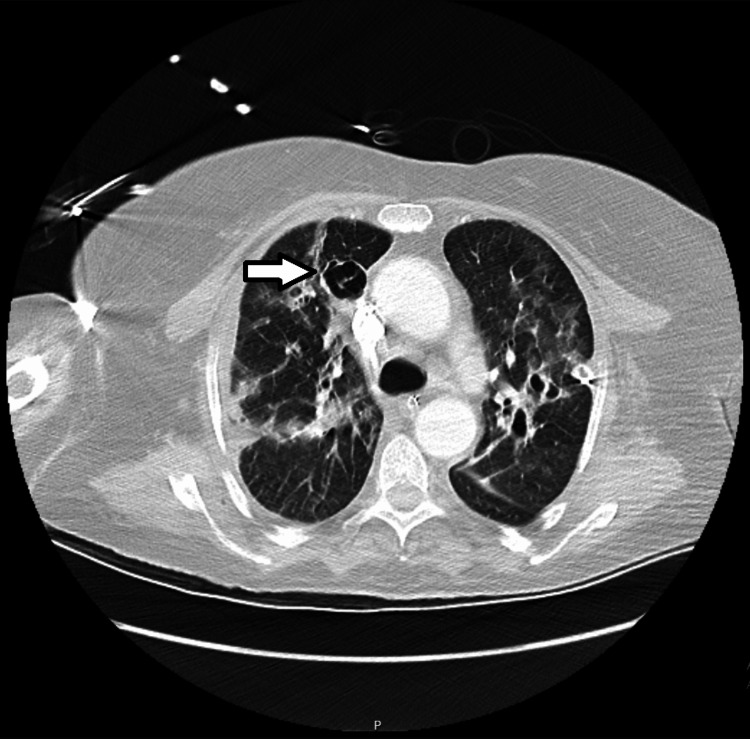
Computed tomographic scan of the thorax on the 20th day in the intensive care unit Diffuse bilateral subpleural ground-glass opacities with fibrotic components and necrotizing bilateral pneumonia (arrow) are evident.

These bilateral hypertensive pneumothoraces developed mainly as a result of ventilation-induced lung injury in association with the parenchymal injury already caused by COVID-19 and were successfully treated with bilateral chest tubes. After the resolution of these complications, on Day 20, fever and new deterioration of ventilatory function led to the diagnosis of necrotizing bilateral pneumonia, as seen in Figure 6, with identification of methicillin-susceptible staphylococcus aureus in microbiology results, requiring antibiotic therapy adjustment.

On Day 27 patient was diagnosed with a perisplenic abscess which was drained with a pigtail catheter. In spite of these complications, there were no other hemorrhagic or thrombotic manifestations, and the patient's status improved slowly day by day. On Day 41 in the ICU, invasive ventilation was finally stopped. On Day 62 the patient was discharged to the internal medicine ward, where she would receive care until Day 87, when she was discharged from the hospital.

## Discussion

As mentioned earlier, patients with COVID-19 can have mild symptoms or severe disease with systemic inflammatory responses; SARS and cytokine storms can lead to organ dysfunction and worsen the prognosis. Such severe acute events are particularly prevalent among elderly individuals and patients with comorbidities such as obesity, hypertension, diabetes, or COPD. Additionally, respiratory viruses can trigger exacerbations of asthma, which can increase the severity of the infection. Coronaviruses have already been implicated as triggers of asthma exacerbations [15]. 

In our patient, slight changes in laboratory values were already identifiable at the time of admission (the 10th day after symptom onset); during the next four days, platelet counts and fibrinogen levels decreased rapidly to extremely low values. Of the laboratory values, those of d-dimers were the last to reflect clinical deterioration. This finding is consistent with that of Asakura and Ogawa, who observed that in patients with severe COVID-19, severe coagulopathy developed during Days 10-14, with large fluctuations in coagulation and fibrinolysis markers between Days 7 and 10, with a sharp increase of fibrin degradation products and d-dimer levels in just three days. However, among survivors, almost no changes in coagulation and fibrinolysis markers occurred during this same period [4]. The hemorrhagic manifestations seen in our patient started at the end of the fourth day of hospitalization, with massive airway bleeding. DIC was diagnosed by the fifth day, which was consistent with the results of Tang et al., who reported that the median time from admission to DIC diagnosis was four days [16].

In our patient, the normalization of the laboratory parameters was slow; thus, DIC did not resolve immediately because the resolution requires several conditions: synthesis of coagulation factors, which are produced at different rates; clearance of anticoagulant factors and fibrin degradation products from the circulation, which depend on hepatic function; and production of new platelets from the bone marrow, which may take several days [6].

From this clinical case and the literature associated with CID in COVID-19, we identified asthma and obesity as risk factors that could have determined the severe progression of COVID-19 in our patient, particularly with the development of such a life-threatening condition as DIC. On the other hand, possibly her being female, combined with the absence of other risk factors and comorbidities, may have played a role in her survival, in contrast to the poor outcome expected with the clinical development of both conditions simultaneously. 

## Conclusions

In this work, we describe COVID-19 as a precursor of DIC, triggering an inflammatory response that leads to cytokine release, although the manifestations differ slightly from those of sepsis-related DIC, according to other studies. The periodic control of coagulation and fibrinolysis parameters is of key importance because rapid changes can occur and lead to hemorrhagic manifestations that further worsen the patient’s clinical condition and prognosis. The diagnosis of DIC is based on clinical and laboratory findings, and its prompt identification is the key to better management of its complications.
